# The influence of interparticle cohesion on rebounding slow impacts on rubble pile asteroids

**DOI:** 10.1038/s41526-022-00221-8

**Published:** 2022-08-17

**Authors:** Kolja Joeris, Laurent Schönau, Matthias Keulen, Philip Born, Jonathan E. Kollmer

**Affiliations:** 1 Experimentelle Astrophysik, Universität Duisburg-Essen, Lotharstr. 1-21, 47057 Duisburg, Germany; 2 Institut für Materialphysik im Weltraum, Deutsches Zentrum für Luft- und Raumfahrt (DLR), 51170 Köln, Germany

**Keywords:** Condensed-matter physics, Physics

## Abstract

The ballistic sorting effect has been proposed to be a driver behind the observed size sorting on the rubble pile asteroid Itokawa. This effect depends on the inelasticity of slow collisions with granular materials. The inelasticity of a collision with a granular material, in turn, depends on grain size. Here we argue that determining the inelasticity of such collisions in an asteroid-like environment is a nontrivial task. We show non-monotonic dependency of the coefficient of restitution (COR) on target particle size using experiments in microgravity. Employing numerical simulations, we explain these results with the growing influence of adhesion for smaller-sized particles. We conclude that there exists an optimum impactor to target particle size ratio for ballistic sorting.

## Introduction

Many asteroids are expected to be rubble piles^1–4^. The evolution and dynamics of such celestial bodies are therefore governed by the mechanics of granular matter^5^. Recent exploration missions have revealed a complex surface topology, in particular size-sorted areas of rocks and regolith on the surface of asteroid Itokawa^6^, giving further consideration to the granular mechanics of such bodies. This size sorted surface is in contrast to the expectation one might have from the random accretion of dust and rocks^7–9^. Thus, a sorting mechanism is required.

Two processes based on the mechanics of dry granular particles were suggested to cause the lateral size segregation process, among others^10^. The Brazil nut effect (BNE) is known to cause size segregation in shaken or vibrated granular media^11–13^. The seismic activity required for such a planetesimal-spanning mobilization of material can be triggered in asteroids by high-velocity impacts onto the weakly bound rubble piles^14,15^. In contrast, the ballistic sorting effect (BSE) effectively guides particles with low velocities to seas of small particles, while keeping larger boulders free of pebbles and grains^16,17^. The BNE and BSE are not necessarily exclusive explanations for the observed surface features on rubble pile asteroids, and a combination of both effects might be at work^18^.

The BSE relies on the difference in the elasticity of low-velocity collisions depending on the target composition. While an impactor bounces off a massive boulder with little dissipation of kinetic energy, it efficiently loses its velocity when impacting a bed of smaller particles^16^. Such low-velocity impacts can either occur during the initial phases of planetesimal formation^19^ or from secondary impacts. Secondary impacts can happen when a high-velocity impactor mobilizes particles from the asteroid surface. The velocity at which the particles mobilized by the primary impact settle again and re-impact the asteroid is limited by the asteroid’s low escape velocity because faster particles will no longer be gravitationally bound. This type of impact has been studied before in low gravity, however, these studies mainly focused on ejecta generation^20–23^ or crater formation^24^. We do not expect our results to scale predictably for very high impact speeds where the impactor carries enough energy to mobilize gravitationally and cohesively bound particles without distinction.

So far, the BSE has only been tested numerically and experimentally for millimeter-sized particles in the gravity-dominated regime, i.e., a regime where gravity dominates over cohesion^16^. Under such conditions, the dissipation of energy during impact depends on the number of mobilized contacts. A bed of small particles provides a higher number of contacts per volume and hence causes stronger dissipation for an impacting particle than a bed of massive boulders.

In size distributions similar to those found in regolith on the moon and other celestial bodies^25^, fine grains overwhelmingly outnumber larger rocks. This means that the majority of collision events involve small particles. Additionally, while large rocks are mostly unaffected by interparticle cohesion due to their high mass, for small particles the force ratio between gravity and cohesion is shifted in favor of the latter. On bodies like Itokawa, the cohesion-dominated regime already starts at centimeter-sized particles due to the low gravity. In order to fully understand size sorting on asteroids this means that we need to investigate the interplay of gravity and cohesion in an environment where both forces approach the same magnitude. Another sign of “non-intuitive” behavior for cohesive granular matter in low gravity is present in the data of Brisset et al.^20^. It already appears to show that impacts in fine JSC-1 regolith simulant tend to be more elastic than impacts on beds of larger quartz sand, counter the expectation for dry cohesionless granular materials as expressed in the preceding paragraph.

This paper is organized as follows: Starting with the introduction above, we first present our experimental results. We revisit the scaling of the dissipation, as described by the coefficient of restitution (COR), during impacts in particle beds with different particle sizes and with different cohesion^26^ to gravitational forces ratios. We perform a series of low gravity impact experiments and complement our experimental results with numerical simulations to monitor the scaling of the COR for impacts into beds of small and cohesive particles. Our results show that the simple scaling of the COR with particle size breaks down for beds of fine regolith particles. The numerical simulations reveal that the observed non-monotonic behavior of the COR is a consequence of inter-particle adhesion. Details on the experimental and numerical setup can be found in the Methods section at the end of the manuscript.

## Results

### Drop tower experiment

The COR is a convenient parametrization of the energy dissipation experienced by the impacting particle. The COR is defined to be the ratio of the velocity before impact *v* and the velocity after rebound *v*′, COR = |*v*′|/|*v*|^27^. We measure the COR by tracking the impactor before and after the collision with the particle bed, yielding the velocities |*v*| and |*v*′| to compute the COR. In cases where the impactor did not rebound, but, for example, was buried beneath the bed surface, we assign a COR of 0.

**Fig. 1:**
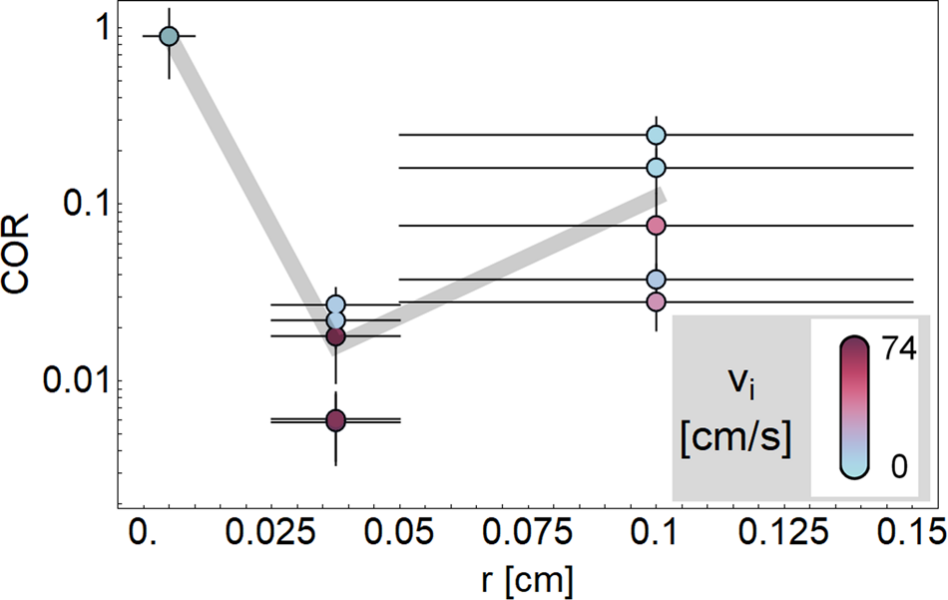
Experimental coefficient of restitution (COR) as function of bed particle radius *r*. All points are obtained in reduced gravity of *g* = 2 × 10^−3^ ± 10^−6^ m/s^2^. Impact velocities are color coded. The velocity range is not equal for all particle sizes due to experimental constraints. Mean values are connected by a gray line to guide the eye. Error bars for the COR-values indicate uncertainties in particle tracking, and for the particle size the width of the particle size distribution as supplied by the manufacturer. Interestingly, the COR first decreases when comparing large (0.05–0.15 cm) to medium (0.025–0.05 cm) sized bed particles, but then is highest for the smallest (<0.01 mm) bed particle size.

Figure 1 shows the results of our drop tower experiments. Our impactors are irregularly shaped basalt particles with a radius of ≈0.15 cm and a mass between 40 and 60 mg. We used three different target material grain sizes: 0.05–0.15 cm irregular basalt, 0.02–50.05 cm irregular basalt, and finely powdered JSC MARS-1 simulant, which was available from a previous experiment. For more details on the drop tower experiment setup and performance see ref. ^28^.

The initial velocity of the impactor thereby varied between 3 and 74 cm/s. Contrary to the assumption in Shinbrot et al.^16^, a monotonic scaling of the COR with bed particle size cannot be confirmed (see Fig. 1). The high level of restitution observed with a bed of very fine particles strongly suggests a change of the scaling of the dissipation. An effect of the different impact velocities on the COR for the different particle beds might still be present, but is dominated by a size effect in the observed parameter range.

### Numerical simulations

The repetition rate and the number of experimental data points are limited by the availability of the drop tower. A finer parameter resolution, especially with respect to the bed particle size, is necessary to test the COR scaling. Numerical impact experiments are employed for this purpose. In those simulations, the bed particle radius *r* was systematically varied between 0.06 and 0.46 cm, while the impact velocity and the microgravity were fixed at 10 cm/s and *g* = 2 × 10^−3^ m/s^2^.

**Fig. 2:**
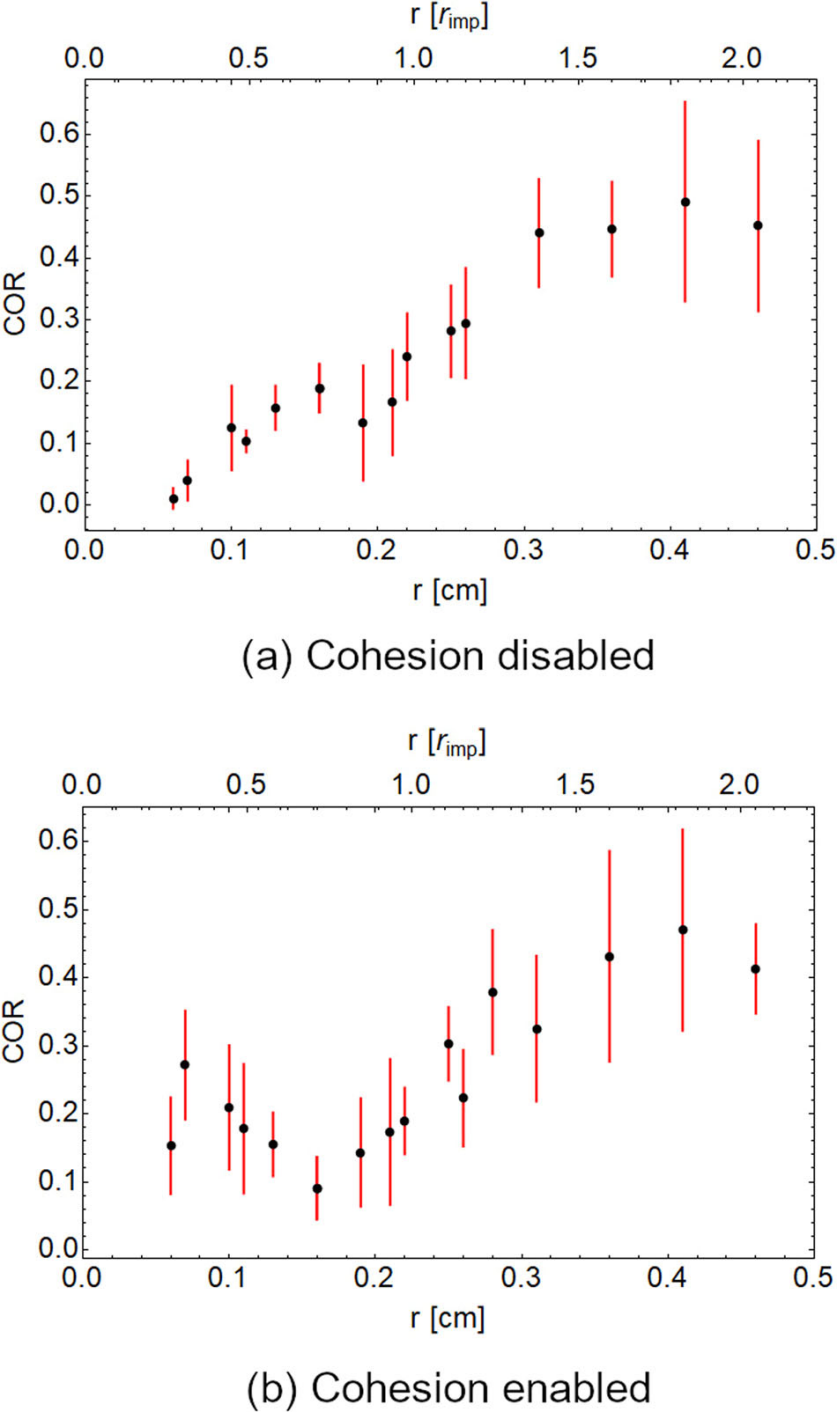
Simulation data for COR as a function of bed particle size. The gravity is set to *g* = 2 × 10^−3^ m/s^2^. **a** Without cohesion, **b** with a cohesion defined b a surface energy of γ = 300 mJ/m^2^. Impact velocity is kept constant at *v* = 10 cm/s. Error bars denote the standard deviation of 5 simulation runs each. **a** Without cohesion, a steady decline of the COR with smaller bed particles is observed. **b** A local minimum appears to be present with cohesive particles for bed particles with a radius of *r* = 0.18 cm.

The results of the impact simulations are summarized in Fig. 2. The numerical data reproduce the expectation for cohesionless granular particles, with a monotonic decrease of the COR with decreasing particle size (see Fig. 2a). However, including a realistic cohesion of 300 mJ/m^2^^29^ among the particles in the simulations induces a more complex dependence of the COR on the particle size. A regime for small particle sizes emerges, where the COR grows with decreasing particle size. A local minimum in the COR emerges with bed particles of 0.18 cm radius. Due to limited CPU time no smaller particles could be simulated in this work. This means we cannot make further assumptions about the COR for systems made of smaller simulated particles.

**Fig. 3:**
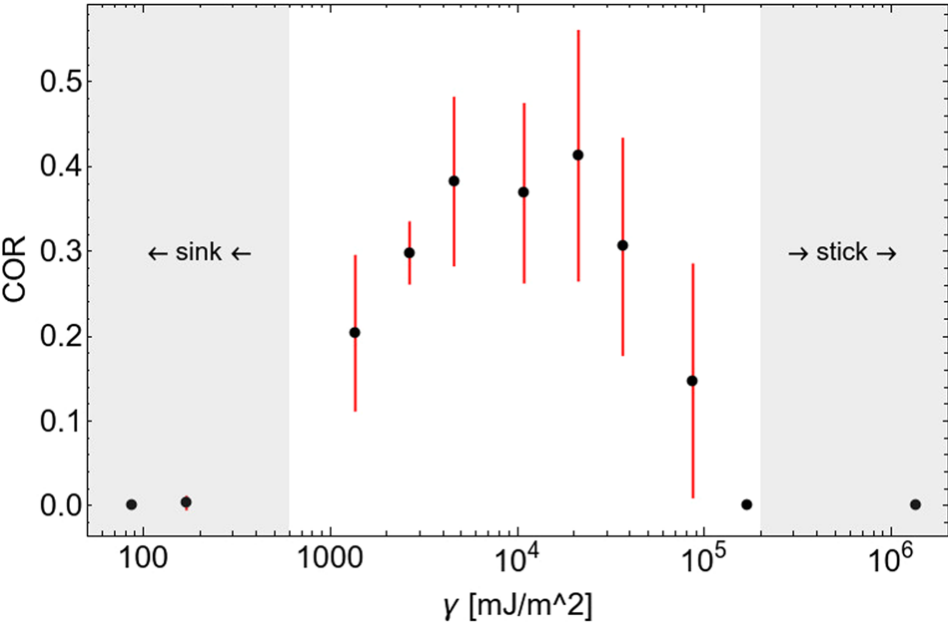
Simulated COR over surface energy γ. All points are obtained with *g* = 2 10^−3^ m/s^2^, bed particle size *r* = 0.06 cm, and an impactor velocity of 10 cm/s. Error bars denote the standard deviation of 5 simulation runs each. The shaded areas emphasize different characteristics of impactor-bed interaction. While the impactor sticks to the particle bed at high surface energies, it sinks into the bed of low-cohesive particles. In between, at moderate surface tensions, a significant enhancement of the COR can be observed.

The particle size and the cohesion can be varied independently in the numerical study. In Fig. 3, we give the result of varying the cohesion among particles of size *r* = 0.6 mm in impact experiments as before. Three different regimes can be discriminated in the COR: 1. The impactor can hit the bed and sink in without rebounding. This sinking regime emerges at sufficiently low cohesion among the bed particles, and can be observed for surface energies γ < 10^3^ mJ/m^2^ in Fig. 3. A significant displacement of bed particles by the impact is observed in this regime, and the impactor comes to rest below the point of the first collision. 2. The cohesion between impactor and particle bed becomes so high that the impactor does not rebound in a sticking regime. For the impact velocities and impactor size in this study, this happens at γ > 10^5^ mJ/m^2^. This value of γ lies far beyond any realistic expectation for basalt or similar materials. In the sticking regime the granular bed is bound by cohesion instead of gravity. The impactor distorts the structure of the bed far less than in the first regime and comes to rest almost immediately, at the point of the first contact. 3. The rebounding regime emerges in between those two extreme regimes. A bed of cohesive granular material can thus provide a range of collisional outcomes depending on the exact value of gravity, cohesion, particle size, and impact velocity.

## Discussion

The microgravity experiment and the numerical results both indicate the same: The COR is a complicated quantity for low-velocity impacts in granular surfaces under conditions of low gravity. This non-monotonic scaling of energy dissipation was observed in a realistic experimental situation where impactors of various velocities hit beds of irregular polydisperse particles, as well as in idealized numerical simulations with spherical grains.

The simulations are a highly idealized replication of the experiment. In particular, particle shape, material properties, initial packing, and size distribution differ from the experiment. The tighter packing of samples with a broad size distribution might amplify the COR scaling in our experiment, still simulations show that a tight packing is not needed to explain the results qualitatively. The observation that both simulation and experiment show a dip in the COR for a certain size ratio suggests that this dip is a universal behavior and a consequence of cohesion as seen in Fig. 2. Further investigation of the influence of material parameters on the exact COR function could lead to even deeper insight into the mechanisms of ballistic sorting.

Based on the monotonic COR-scaling assumed for ballistic sorting, all impacting particles, regardless of their size, should aggregate at places that initially were covered with small particles. Our result now shifts this assumption. In particular, it implies that the sorting depends on substrate particle size and cohesivity. Further studies should address the question on whether the impactor to bed particle size ratio for optimal dissipation also scales with impactor size and/or velocity. The results presented here may hint to a bias of ballistic sorting towards slowly impacting particles being most efficiently captured by beds of particles with similar or slightly smaller size. Observing such a biased sorting structure would be strong evidence for ballistic sorting. Producing high-resolution surface maps to determine such a possible bias the in sorting efficiency of centimeter-sized and smaller objects would be a challenge for future asteroid exploration missions. Similarly, a higher experimental resolution of CORs, especially for low-speed impact on fine powders, could complete the understanding we have of sorting mechanisms.

## Methods

Impacts on asteroid surfaces were experimentally and numerically simulated. Both simulations of the asteroid surface included the gravity and the vacuum existing on an asteroid.

### Experiments

**Fig. 4:**
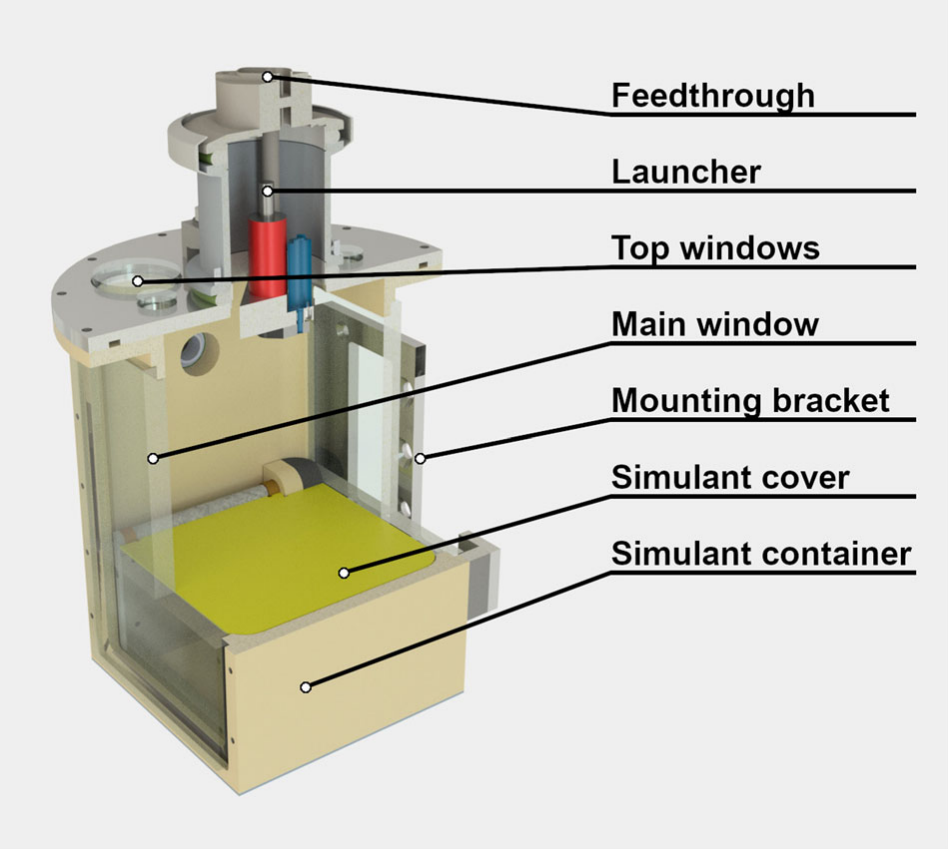
Schematic rendering of the experiment vacuum chamber. The launcher for the impactor and the particle bed is situated inside the chamber, while the hole chamber is mounted to a high-precision linear stage to produce the desired microgravity during a drop tower flight.

The small gravity existing on an asteroid is simulated by linearly accelerating a particle bed under minimal gravity. The ZARM drop tower (Bremen, Germany) provides residual accelerations below 10^−6^*g* inside the drop capsule^30^. We do not use a centrifuge to generate the artificial gravity to avoid the coriolis forces the impactor would experience when traversing the experiment chamber^31^. Instead, a high precision linear stage carries a vacuum chamber inside the drop capsule. The experimental vacuum chamber is sketched in Fig. 4. Its top section is comprised of a launcher mechanism, which is able to accelerate impactors to cm/s velocities, and its lower section includes the target material mimicking the asteroid surface, shielded by a motorized cover for launch and landing. A vacuum level of below 0.1 mBar is achieved by running a turbo vacuum pump until shortly before launch. Three cameras are used to observe the granular system, one through the main window at the side and two through smaller windows on the top.

The experimental procedure followed during the 9.3 s of microgravity during a catapult flight in the drop tower consists of several stages: during the first second, any residual motion from the apparatus is allowed to decay. Then the vacuum stage is accelerated for one second five times stronger than the desired asteroid gravity to settle any material that might have been become loose during launch. The last second of the flight is dedicated to mechanically securing the experiment for the high-*g* levels of the landing. This leaves a usable observation time of ≈5 s. During this time, the acceleration is tuned to *g* = 2 × 10^−3^ ± 10^–6^ m/s^2^. This gravity level corresponds to a body with a diameter of 2.6 km and a mass of 5 × 10^13^ kg. Surface gravities of that magnitude would be found on asteroids with dimensions similar to, for example, 4179 Toutatis. It is chosen to accommodate full settling of particle bed movements within the available time, while still keeping all used particle sizes in the cohesion-dominated regime. The impactors are irregularly shaped basalt particles with a radius of *r*_i_ < 0.15 cm and a mass between 40 and 50 mg. We used three different bed particles: 0.5–1 mm irregular basalt particles, 1–3 mm irregular basalt particles, and we used JSC MARS-1^32^ simulant as our fine target material, which was readily available from a previous experiment. For technical details of the drop tower experiment setup and its performance see ref. ^28^.

### Numerical setup

The particle bed dynamics were simulated using the open source DEM software LIGGGHTS 3.7^33^. The simulation box horizontally spans 1.5 cm × 1.5 cm with periodic boundary conditions. The spherical monodisperse particles are poured randomly into this volume, until a filling height of approximately 3 cm is reached. A spherical impactor with radius *r*_i_ = 2.25 mm is inserted just above the bed with a velocity of 10 cm/s. Gravity is set to *g*_a_ = 2 × 10^−3^ m/s^2^. Contacts between beads are computed using a dissipative Hertzian model^34^ as implemented in LIGGGHTS 3.7. The linearized Johnson–Kendall–Roberts model is used as a numerically inexpensive cohesion formula^26^. The contact imposes an additional force of the form *F*_coh_ = 4γπδ*nr*^∗^, with surface energy density γ, normal overlap δ*n*, and reduced radius *r*^∗^. The cohesion is set to γ = 300 mJ/m^2^ if not denoted otherwise. The cohesion is calibrated using the dynamic pull-off force as in ref. ^35^. Simulation parameters are furthermore set to a Young’s-modulus of 1 GPa, Poissons ratio 0.25, coefficient of friction 0.2, and restitution parameter 0.5. The simulated particles are softer than the basalt particles in the experiment. The short contact times of the rigid basalt particles with high Young’s-modulus would necessitate extremely fine simulated time steps resulting in a computation time beyond our capabilities.

### Reporting summary

Further information on research design is available in the Nature Research Reporting Summary linked to this article.

## Supplementary information

Reporting Summary

## Data Availability

All data necessary to reproduce the plots and findings in this paper are available from the corresponding author of this paper on reasonable request.
