# Supplementary material for: The influence of interparticle cohesion on rebounding slow impacts on rubble pile asteroids
Source: NPJ Microgravity. 2022 Aug 17;8:36. doi: 10.1038/s41526-022-00221-8 (PMC9385662; doi:10.1038/s41526-022-00221-8)
Supplement: Supplementary file 1 — Reporting Summary [file 41526_2022_221_MOESM1_ESM.pdf]

nature portfolio

Corresponding author(s): Kolja Joeris
Last updated by author(s): Mar 31, 2022

Reporting Summary

Nature Portfolio wishes to improve the reproducibility of the work that we publish. This form provides structure for consistency and transparency in reporting. For further information on Nature Portfolio policies, see our Editorial Policies and the Editorial Policy Checklist.

Statistics

For all statistical analyses, confirm that the following items are present in the figure legend, table legend, main text, or Methods section.

n/a	Confirmed	
☒	☐	The exact sample size (n) for each experimental group/condition, given as a discrete number and unit of measurement
☒	☐	A statement on whether measurements were taken from distinct samples or whether the same sample was measured repeatedly
☒	☐	The statistical test(s) used AND whether they are one- or two-sided<br>*Only common tests should be described solely by name; describe more complex techniques in the Methods section.*
☒	☐	A description of all covariates tested
☒	☐	A description of any assumptions or corrections, such as tests of normality and adjustment for multiple comparisons
☒	☐	A full description of the statistical parameters including central tendency (e.g. means) or other basic estimates (e.g. regression coefficient) AND variation (e.g. standard deviation) or associated estimates of uncertainty (e.g. confidence intervals)
☒	☐	For null hypothesis testing, the test statistic (e.g. F, t, r) with confidence intervals, effect sizes, degrees of freedom and P value noted<br>*Give P values as exact values whenever suitable.*
☒	☐	For Bayesian analysis, information on the choice of priors and Markov chain Monte Carlo settings
☒	☐	For hierarchical and complex designs, identification of the appropriate level for tests and full reporting of outcomes
☒	☐	Estimates of effect sizes (e.g. Cohen's d, Pearson's r), indicating how they were calculated

Our web collection on statistics for biologists contains articles on many of the points above.

Software and code

Policy information about availability of computer code

Data collection	LIGGGHTS 3.7
Data analysis	Wolfram Mathematica 12.1

For manuscripts utilizing custom algorithms or software that are central to the research but not yet described in published literature, software must be made available to editors and reviewers. We strongly encourage code deposition in a community repository (e.g. GitHub). See the Nature Portfolio guidelines for submitting code & software for further information.

Data

Policy information about availability of data

All manuscripts must include a data availability statement. This statement should provide the following information, where applicable:

- Accession codes, unique identifiers, or web links for publicly available datasets
- A description of any restrictions on data availability
- For clinical datasets or third party data, please ensure that the statement adheres to our policy

All data necessary to reproduce the plots and findings inthis paper are available from the corresponding author oft his paper on reasonable request.

Field-specific reporting

Please select the one below that is the best fit for your research. If you are not sure, read the appropriate sections before making your selection.

☐ Life sciences ☐ Behavioural & social sciences ☒ Ecological, evolutionary & environmental sciences

For a reference copy of the document with all sections, see nature.com/documents/nr-reporting-summary-flat.pdf

Ecological, evolutionary & environmental sciences study design

All studies must disclose on these points even when the disclosure is negative.

Study description	The coefficient of restitution of a basalt impactor was measured for different materials and velocities under asteroid conditions. The experiment was replicated using numerical simulations.
Research sample	The research sample contains all impacts we could produce during our drop tower campaigns.
Sampling strategy	Due to the nature of this kind of experiment and the low number of data point no sampling strategy is needed, we use all data points we could acquire.
Data collection	The experimental data was recorded automatically via high-speed cameras for each experiment run.
Timing and spatial scale	All data was taken from drop tower experiments in the Bremen drop tower in the years 2019 and 2020.
Data exclusions	No data from succesfull experiment runs were excluded.
Reproducibility	The environment was controlled as precisely as possible, with additional hall sensors measuring the chamber displacement to verify the experiments performance, accelerometers in the drop tower capsule and using pressure sensors to verify the asteroid environment. Those data were logged when possible.
Randomization	The granular bed is categorized by particle size, but in the terms of a statistical analysis (which is not the scope of our paper) this does not seem to be relevant.
Blinding	In our work we don't deal with participants like in clinical studies, so any type of blinding related to those does not apply. The impactor velocity was not exactly predetermined for each examined and only measured during the analysis, resulting in a sort of blinding possibly reducing experimenters bias.

Did the study involve field work? ☐ Yes ☒ No

Reporting for specific materials, systems and methods

We require information from authors about some types of materials, experimental systems and methods used in many studies. Here, indicate whether each material, system or method listed is relevant to your study. If you are not sure if a list item applies to your research, read the appropriate section before selecting a response.

Materials & experimental systems

n/a	Involved in the study	
☒	☐	Antibodies
☒	☐	Eukaryotic cell lines
☒	☐	Palaeontology and archaeology
☒	☐	Animals and other organisms
☒	☐	Human research participants
☒	☐	Clinical data
☒	☐	Dual use research of concern

Methods

n/a	Involved in the study	
☒	☐	ChIP-seq
☒	☐	Flow cytometry
☒	☐	MRI-based neuroimaging
